# Is the Effect of Aerobic Exercise on Cognition a Placebo Effect?

**DOI:** 10.1371/journal.pone.0109557

**Published:** 2014-10-07

**Authors:** Cary R. Stothart, Daniel J. Simons, Walter R. Boot, Arthur F. Kramer

**Affiliations:** 1 Department of Psychology, Florida State University, Tallahassee, Florida, United States of America; 2 Psychology Department, University of Illinois at Urbana-Champaign, Champaign, Illinois, United States of America; University of California, San Francisco, United States of America

## Abstract

A number of studies and meta-analyses conclude that aerobic fitness (walking) interventions improve cognition. Such interventions typically compare improvements from these interventions to an active control group in which participants engage in non-aerobic activities (typically stretching and toning) for an equivalent amount of time. However, in the absence of a double-blind design, the presence of an active control group does not necessarily control for placebo effects; participants might expect different amounts of improvement for the treatment and control interventions [1]. We conducted a large survey to explore whether people expect greater cognitive benefits from an aerobic exercise intervention compared to a control intervention. If participants expect greater improvement following aerobic exercise, then the benefits of such interventions might be due in part to a placebo effect. In general, expectations did not differ between aerobic and non-aerobic interventions. If anything, some of the results suggest the opposite (e.g., respondents expected the control, non-aerobic intervention to yield bigger memory gains). These results provide the first evidence that cognitive improvements following aerobic fitness training are not due to differential expectations.

## Introduction

Aerobic exercise interventions lead to improved cognitive performance. Relative to participants in control groups, people who engage in aerobic exercise (typically walking) show improved executive control and memory, as well as enhanced spatial abilities and processing speed [2] [3] [4] [5]. Intervention studies can provide direct support for the causal efficacy of aerobic exercise, bypassing third-variable and directionality criticisms associated with cross-sectional or correlational studies of the link between aerobic fitness and cognition.

The strength of the evidence for the causal potency of aerobic exercise must be evaluated relative to the control groups used in these interventions. Encouragingly, many such studies include an active control group that engages in some form of physical activity other than aerobic exercise. Compared to passive, no-contact, or “sit-and-wait” control groups, active control groups provide a better check against placebo effects; doing *something* likely induces greater expectations for improvement than doing nothing.

Yet, even the use of an active control group may not fully equate expectations for improvement. To conclude that the improvements were caused by the critical ingredient in the intervention, the control group must equate for all aspects of the intervention other than the critical ingredient, including any expectations induced by the intervention itself. Yet, most “brain training” intervention studies do not equate the training and control interventions on additional factors and almost none control for the possibility of differential expectations [1]. Unfortunately, without equating expectations in the experimental and control group, any differential improvements might be due to placebo effects.

One way to determine whether differential expectations might drive improvements is to measure expectations for improvement in a separate group of participants. For example, in the video game training literature, those in the experimental group typically play an action video game like Unreal Tournament, and those in the control group typically play a game like Tetris or The Sims. When participants were first shown one of these games and then asked to judge whether playing the game would enhance performance on various measures of visual processing, attention, spatial ability, and memory, the pattern of expected improvement matched the pattern of observed improvement in the literature [1]. In other words, the pattern of improvements in the literature on video game interventions is consistent with a placebo effect. Without controlling for such expectation differences, researchers cannot conclude that these interventions have causal potency above and beyond the expectations they induce.

Could similar expectation effects undermine conclusions about the causal potency of aerobic exercise in improving cognition? We report the results of a survey in which participants first learned about either an aerobic or non-aerobic intervention and then judged how much the intervention would improve performance on each of several cognitive tasks commonly used in such interventions. If people expect greater improvement from aerobic exercise than from the typical stretching and toning control intervention, then the results would be consistent with a placebo effect. If people have roughly comparable expectations for improvement or if their expectations favor the stretching and toning condition, then placebo effects are unlikely to drive the cognitive gains from aerobic exercise.

## Method

This study was pre-registered at the Open Science Framework (https://osf.io/brmvq/) where we provide descriptions of the testing plan, exclusion criteria, and stopping rules.

### Participants

Participants were recruited and tested online using Amazon Mechanical Turk. To be able to participate in the experiment, participants needed an MTurk approval rating of 90% or higher and had to be living in the United States (approval rating is the percentage of times that an MTurk worker has successfully received credit for participating in a study out of the total number of times they have participated–it reflects their quality of work across studies). In total, 657 participants were recruited for the study. To ensure that only the highest quality data were included in our analyses, we pre-specified strict inclusion criteria that excluded from analysis data from participants who provided incomplete answers (*n* = 235) and those who either misunderstood the task or reported having knowledge of the link between aerobic exercise and cognition (*n* = 251). Task comprehension was independently judged by 2 coders, and participants were excluded if either coder thought that they should be. This strict exclusion process was designed to ensure that participants fully understood the nature of the described intervention and each cognitive measure. The final data set included 171 participants, with 72 in the non-aerobic group (40 females, mean age = 35.15, *SD* = 13.11) and 99 in the aerobic group (47 females, mean age = 34.17 years, *SD* = 12.95). Participants were paid $0.25 for completing the study. This study was approved by the Institutional Review Board at the University of Illinois at Urbana-Champaign, with a waiver of the requirement for signed consent due to the anonymous nature of the survey (participants read a consent screen before participating).

## Materials and Procedure

Participants read about one of two exercise interventions (see Table 1): walking 3 times a week for up to 40 minutes each time (aerobic) or doing stretching, toning, and resistance training 3 times a week for 50 minutes each time (nonaerobic). On a separate screen, and without being able to view the intervention description again, participants were then asked to briefly summarize what they had just read about the intervention. Participants then read about and watched videos depicting three cognitive tasks commonly used in studies of the benefits of exercise: a task switching task (similar to the one used by [6], see Video S1), a relational memory task (similar to [7]; see Video S2), and a reaction time task (see Video S3). The reaction time task was included because fitness effects appear to be smaller for this type of task compared to the others and it provides for an opportunity to assess differential expectations, not only for training, but for outcome measures [2]. After each video and task description, participants were asked: (1) if they believed that completing the exercise intervention they read about earlier would improve performance on the task, and if so, how much (6 item Likert-type scale ranging from “a little” to “a lot”); and (2) to summarize the task on a separate screen without the ability to view the description of the task or its video again. Participants were then asked if they have ever read or heard about research showing that exercise can improve cognition, and if so, what they had read or heard. Finally, participants provided demographic information and answered questions about their exercise habits. A PDF version of the survey is available online under Text S1.

**Table 1:** The intervention and task descriptions that participants read.

Task/Intervention	Description Providedto Participants
AerobicIntervention	Think about the following fitness training procedure thathas been used with elderly, sedentary (not fit and not active)adults. The training process takes place three times each week,lasts for a total of 6 months, and involves walking regularly.During the first week, participants walk for 10 minutes onthree different days. Each week, they walk for 5 additionalminutes each day. So, in the second week, they walk for15 minutes each day. For the third week, they walk for20 minutes each day. They continue increasing the timespent walking by 5 minutes each week until, during the7th week, they reach a maximum of 40 minutes of briskwalking three times each week. They continue walking40 minutes three times each week through the rest ofthe 6­month training. Each session included a total of10 minutes of stretching to warm up and cool down.
Non-AerobicIntervention	Think about the following fitness training procedurethat has been used with elderly, sedentary (not fit andnot active) adults. The training process takes placethree days each week, lasts for a total of 6 months,and involves stretching, toning, and balance exercises.During each 50­minute session, participants completethe following: (a) four muscle toning exercises usinghand­held weights or resistance bands participants,(b) two exercises to increase balance, (c) one yogasequence, and (d) one stretching/toning exercise oftheir choice. Every three weeks, participants learn anew set of exercises. In the first week, they learnthe new exercises. During the second and thirdweeks, they try to increase the intensity by addingweight or repetitions of the exercise. The fullprotocol lasted for 6 months, and each sessionincluded 40 minutes of exercise and a total of10 minutes of stretching to warm up and cool down.
ReactionTimeTask	This task measures reaction time: How quickly cansomeone respond to simple events? The primarymeasure of performance is speed. On each trial ofthis task, a green square appears either on the leftor right side of the screen. If the square appearson the left, the participant presses the “z” key ontheir keyboard. If the square appears on the right,the participant presses the “/” key on their keyboard.They are asked to respond as quickly as possible
RelationalMemoryTask	This is a memory task that measures the ability toremember the relationship between two pieces ofinformation. Specifically, it tests whether peopleremember if the two pieces of information occurredtogether. Participants first study pairs of imagesand later are tested on their memory for therelationship between them. The primary measureof their performance is memory accuracy. Oneach trial of the study phase, a photograph of ascene is paired with a photograph of a face. Eachscene has one face that appears in a window infront of it. The participant's task is to view eachscene­face pair and to remember which faceappeared with which scene. During the test phase,they again view scene­face pairs and have todecide whether that face had appeared with thatparticular scene during the study phase.
TaskSwitchingTask	This task measures how quickly and accurately someonecan keep two different tasks in mind and switch backand forth between them. The primary measure ofperformance is the cost of having to switch betweentasks. That is, how much slower are people whenthey have to switch tasks compared to when theykeep doing the same task? On each trial of this task,participants view a series of numbers and make oneof two judgments about each one. The judgmentthey have to make depends on the color of thebackground behind the number. If the numberappears against a blue background, participantsjudge whether it is greater than 5 or less than 5.If the background is pink, they judge whether thenumber is odd or even. They respond using the “z”key and the “/” key on their keyboard. The taskcompares their response speed when the have tomake the same response two trials in a row to theirresponse speed when they have to switch whichresponse they are making.

## Results

All analyses were conducted using R version 2.15.1, and the data (Data S1) and analysis script (Text S2) are available online. All analyses presented here were planned and were pre-registered online at https://osf.io/brmvq/. We conducted two sets of analyses. The first included all participants and the second included only participants who reported that they did not exercise. This subgroup of participants matches the typical sedentary population recruited for exercise intervention studies. The proportion of participants expecting improvement was assessed using a chi-square test for the complete data set and with a Fisher’s exact test for the sedentary subset (in each analysis, at least one cell had a count of less than 5). The amount of improvement participants expected was assessed using t-tests. Odds ratios less than 1 (for the chi-square/Fisher’s exact tests) and positive mean differences (for the t-tests) indicate higher values (either means or proportions) for the non-aerobic group.

### Expected Improvement-All Participants

**Figure 1:**
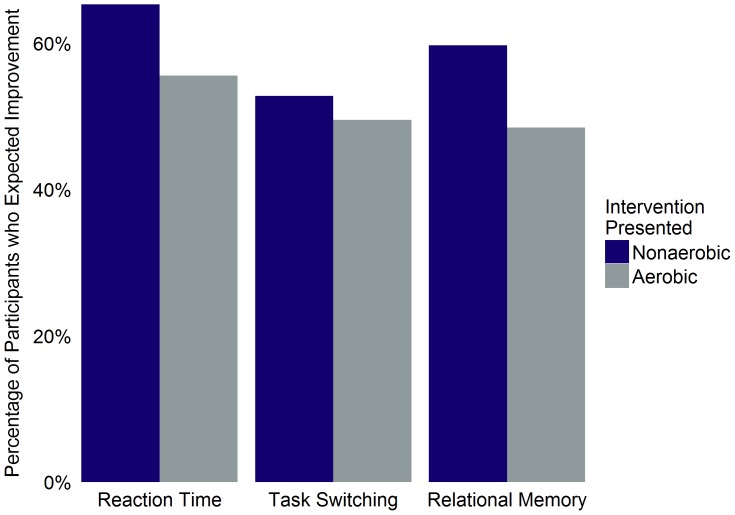
Percentage of participants expecting improvement as a function of intervention group and task for all participants. Percentage of participants within each intervention group who believed that completion of the intervention presented to them would improve task performance. Includes both sedentary and non-sedentary participants.

The proportion of participants who believed that the exercise intervention would improve cognitive performance did not differ significantly between the aerobic and non-aerobic intervention groups for any of the tasks (reaction time: *X* ^2^(1) = 1.64, *OR* = 0.67, 95% CI [0.35, 1.24], *p* = .20; task switching: *X* ^2^(1) = 0.18, *OR* = 0.88, 95% CI [0.48, 1.61], *p* = .67; relational memory: *X* ^2^(1) = 2.11, *OR* = 0.64, 95% CI [0.34, 1.17], *p* = .15; Figure 1).

**Figure 2:**
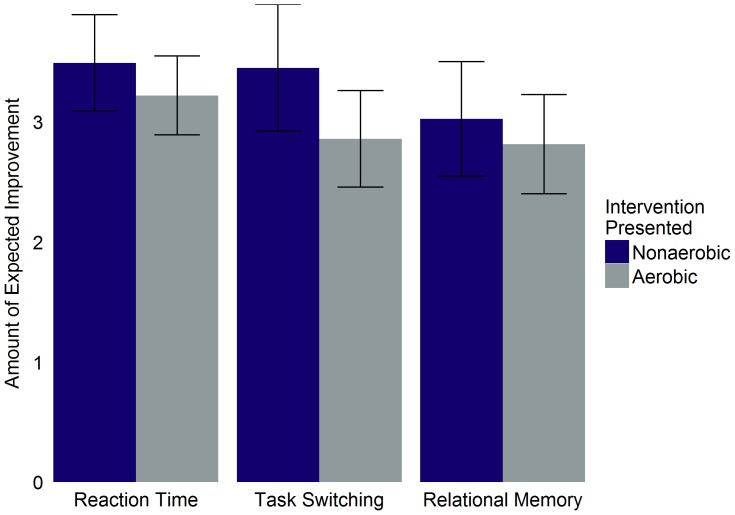
Believed improvement amount as a function of intervention group and task type for all participants. Mean amount of improvement participants believed completion of the intervention presented to them would create. Includes both sedentary and non-sedentary participants. Error bars represent 95% confidence intervals.

For those participants who believed that the intervention would improve performance of a given cognitive task, the amount of improvement they expected did not differ significantly between the aerobic and non-aerobic intervention groups (reaction time: *t*(100) = 1.06, 95% CI [−0.24, 0.78], *p* = .29; task switching: *t*(85) = 1.82, 95% CI [−0.05, 1.23], *p* = .07; relational memory: *t*(89) = 0.68, 95% CI [−0.41, 0.83], *p* = .50; Figure 2).

### Expected Improvement-Sedentary Participants

**Figure 3:**
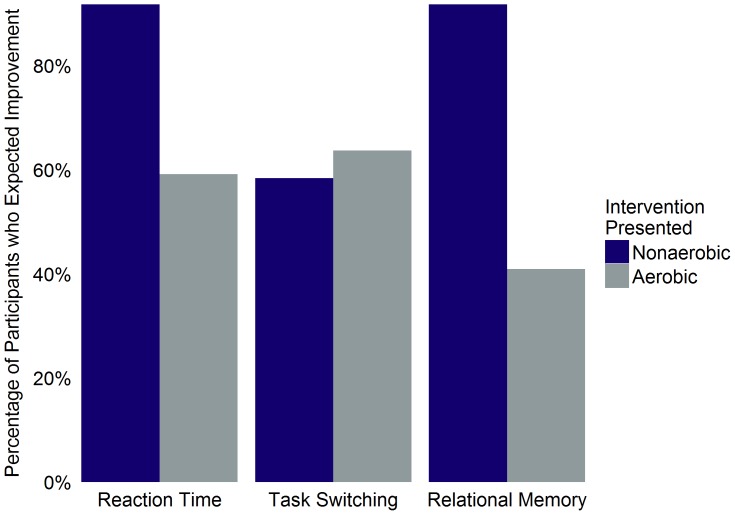
Percentage of participants expecting improvement as a function of intervention group and task for sedentary participants. Percentage of participants within each intervention group who believed that completion of the intervention presented to them would improve task performance. Includes only sedentary participants.

Restricting our analyses to the subset of 34 sedentary participants (non-aerobic: *n* = 12, 6 females, mean age = 41.33, *SD* = 15.47; aerobic: *n* = 22, 10 females, mean age = 36.50, *SD* = 12.38), the proportion of participants who believed that the intervention would improve cognitive performance did not significantly differ between intervention groups for the reaction time task, *OR* = 0.14, 95% CI [0.003, 1.282], *p* = .06, or task switching task, *OR* = 1.24, 95% CI [0.23, 6.52], *p* = 1 (Figure 3). However, a greater proportion of participants assigned to the non-aerobic group expected the intervention to improve performance on the relational memory task, *OR* = 0.07, 95% CI [0.001, 0.617], *p* = .01.

**Figure 4:**
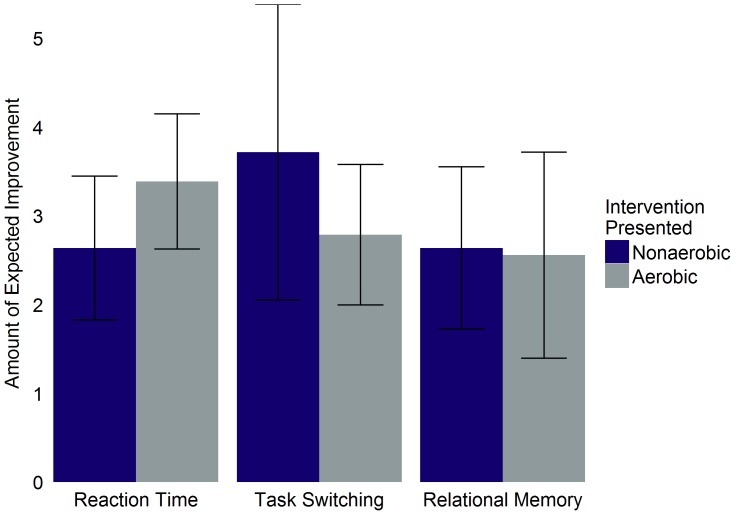
Believed improvement amount as a function of intervention group and task type for all participants. Mean amount of improvement participants believed completion of the intervention presented to them would create. Includes only sedentary participants. Error bars represent 95% confidence intervals.

For those participants who believed that the intervention would improve performance of a given cognitive task, the amount of expected improvement did not significantly differ between intervention groups (reaction time: *t*(22) = −1.48, 95% CI [−1.80, 0.30], *p* = .15; task switching: *t*(19) = 1.32, 95% CI [−0.54, 2.40], *p* = .20; relational memory: *t*(18) = 0.13, 95% CI [−1.27, 1.43], *p* = .90; Figure 4).

## Discussion

Most interventions in psychology cannot use a double blind design because it is not possible to disguise the nature of the intervention: Participants in a video game intervention know what game they are practicing, and participants in an exercise intervention know what exercises they are performing [1]. Double blind designs help to eliminate the possibility that different expectations for improvement underlie differences between the treatment group and the control group (i.e., a placebo effect). In the absence of blinding, different expectations for improvement on each outcome measure between the treatment and control group undermines any inferences about the effectiveness of the treatment-any difference could be driven by a placebo effect. In the case of action video game training, expectations for improvement align nearly perfectly with actual improvements, so claims that training improves cognition may be unwarranted. In contrast, we present initial evidence that differential expectations do not account for improved cognitive abilities following aerobic exercise interventions.

Our survey participants expected roughly comparable cognitive improvements for the aerobic and non-aerobic interventions. Moreover, for individual tasks, those in the non-aerobic group tended to expect greater improvement, with the largest differences in expectations for the task switching difference for all participants, the reaction time difference for sedentary participants, and the relational memory difference for sedentary participants (this last difference in expectations was statistically significant). The differences in expectations for the intervention groups across these tasks could reflect sampling error or it could indicate a correspondence between that task and components of the exercise interventions. For example, because the non-aerobic intervention involved learning and remembering a new set of exercises every three weeks, participants in that group might have expected somewhat greater improvements in relational memory. Overall, this pattern of expectation effects is ideal: People expect the control group to improve more than the treatment group, so greater improvements in the treatment group following an actual intervention are inconsistent with a differential placebo effect.

These are the first data to address the possibility that expectation effects might contribute to improvements in exercise interventions, but several other issues deserve consideration before eliminating the possibility that expectation-driven placebo effects contribute to larger improvements following aerobic exercise interventions. First, the amount of expected improvement was only measured for those who answered “yes” to a question asking if they thought the intervention would lead to improvements. Consequently, we had less power to find differences in the strength of expectations. But, even if we conservatively treat a “no” response as a belief that there would be zero improvement following an intervention, the pattern of results does not change.

Second, in our survey, participants knew about only one of the exercise interventions (i.e., those who learned about the aerobic intervention did not know that other participants had learned about a non-aerobic intervention, and vice versa). In actual intervention studies, participants might not be blind to the existence of other intervention groups. If participants receiving an aerobic fitness intervention know that other participants are receiving non-aerobic training, they might expect greater improvement from their own intervention due to their ability to compare the two interventions. Third, long-term exposure to a treatment might induce different expectations than just reading about that treatment. For example, participants assigned to an aerobic intervention might experience different physiological changes (e.g., more weight loss and lower blood pressure) than those assigned to a non-aerobic intervention, and those changes could result in differential expectations. Future studies could provide short- or long-term interventions in order to determine whether expectations change with exposure. And, intervention studies can and should assess expectations after the study as well (see [8] [9] [10] for recent examples in the cognitive training and gaming literatures).

Although our survey method does not measure all of the ways in which people might develop differential expectations for improvement, the method has proven sufficiently powerful to identify differential expectation effects. Participants who completed a survey much like this one, focusing on video game training interventions, found differential expectation effects consistent with the pattern of actual intervention outcomes [1]. That pattern, unlike the one reported here, is troublesome for the interference that gaming enhances cognition because the difference between conditions is consistent with a placebo effect. In sum, we showed that differential expectations for improvement are unlikely to drive the actual cognitive improvements that follow aerobic exercise interventions.

## Supporting Information

Data S1 **The dataset used for the analyses.**
(TXT)

Text S1 **A PDF version of the online survey.**
(PDF)

Text S2 **The R code used for the analyses.**
(R) Click here for additional data file.

Video S1 **The task switching video shown to participants.**
(MP4)

Video S2 **The relational memory video shown to participants.**
(MP4)

Video S3 **The reaction time video shown to participants.** Note the video erroneously displayed an aquamarine stimulus whereas the instructions referred to a green stimulus. Only 6% of participants reported this discrepancy, so it is unlikely to have influenced expectation judgments.
(MP4)
