# Supplementary material for: Is the Effect of Aerobic Exercise on Cognition a Placebo Effect?
Source: PLoS One. 2014 Oct 7;9(10):e109557. doi: 10.1371/journal.pone.0109557 (PMC4188819; doi:10.1371/journal.pone.0109557)
Supplement: Text S1 — A PDF version of the online survey. (PDF) [file pone.0109557.s002.pdf]

In this HIT, you first will read about a fitness training plan and then you will learn about a task that is commonly used in cognitive psychology. You will be asked to judge how that cognitive task might be affected by fitness training. Finally, we will ask you a few demographic questions.

Think about the following fitness training procedure that has been used with elderly, sedentary (not fit and not active) adults. The training process takes place three times each week, lasts for a total of 6 months, and involves walking regularly.

During the first week, participants walk for 10 minutes on three different days. Each week, they walk for 5 additional minutes each day. So, in the second week, they walk for 15 minutes each day. For the third week, they walk for 20 minutes each day. They continue increasing the time spent walking by 5 minutes each week until, during the 7th week, they reach a maximum of 40 minutes of brisk walking three times each week. They continue walking 40 minutes three times each week through the rest of the 6-month training. Each session included a total of 10 minutes of stretching to warm up and cool down.
Respondents: 50%
Think about the following fitness training procedure that has been used with elderly, sedentary (not fit and not active) adults. The training process takes place three days each week, lasts for a total of 6 months, and involves stretching, toning, and balance exercises.

During each 50-minute session, participants complete the following: (a) four muscle toning exercises using hand-held weights or resistance bands participants, (b) two exercises to increase balance, (c) one yoga sequence, and (d) one stretching/toning exercise of their choice.

Every three weeks, participants learn a new set of exercises. In the first week, they learn the new exercises. During the second and third weeks, they try to increase the intensity by adding weight or repetitions of the exercise. The full protocol lasted for 6 months, and each session included 40 minutes of exercise and a total of 10 minutes of stretching to warm up and cool down.
Respondents: 50%

Briefly summarize the exercise plan you just read about. Make sure to identify the specific exercise(s) described in the plan. Please answer as best you can, even if you are not 100% certain.

Next you will learn about several tasks used in cognitive psychology experiments. For each, we will show yo a description of the task along with a video showing a person performing that task. After you watch it, we will ask you to summarize what you learned. We then will ask you to judge how much better people would perform that task after having engaged in the described fitness plan for 6 months. The next page will give you information about the first task.

This task measures reaction time: How quickly can someone respond to simple events? The primary measure of performance is speed.

On each trial of this task, a green square appears either on the left or right side of the screen. If the square appears on the left, the participant presses the "z" key on their keyboard. If the square appears on the right, the participant presses the "/" key on their keyboard. They are asked to respond as quickly as possible.

Watch this video of a participant completing the task:

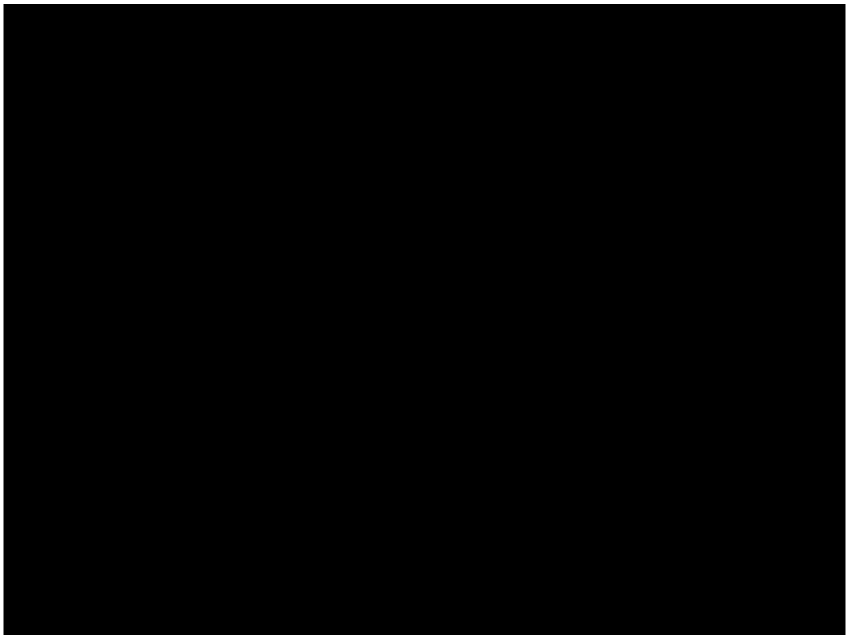

Briefly summarize the cognitive task you just learned about. Make sure to identify what a participant in that task must do and how their performance is measured. Please answer as best you can, even if you are not 100% certain.

Do you think that completing the fitness plan you read about would lead to better performance on this task?

- ○ Yes
- ○ No

If YES, how much improvement do you think would occur?

- ○ 1 (a little)
- ○ 2
- ○ 3
- ○ 4
- ○ 5
- ○ 6 (a lot)

This is a memory task that measures the ability to remember the relationship between two pieces of information. Specifically, it tests whether people remember if the two pieces of information occurred together. Participants first study pairs of images and later are tested on their memory for the relationship between them. The primary measure of their performance is memory accuracy.

On each trial of the study phase, a photograph of a scene is paired with an photograph of a face. Each scene has one face that appears in a window in front of it. The participant's task is to view each scene-face pair and to remember which face appeared with which scene. During the test phase, they again view scene-face pairs and have to decide whether that face had appeared with that particular scene during the study phase.

Watch this video of a participant completing a few trials from the study and test phases of this task:

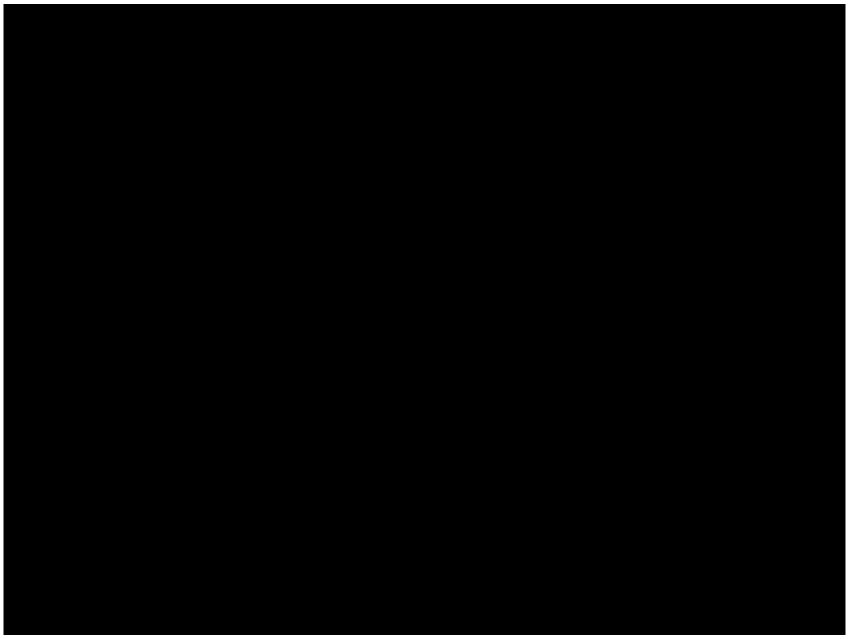

Briefly summarize the cognitive task you just learned about. Make sure to identify what a participant in that task must do and how their performance is measured. Please answer as best you can, even if you are not 100% certain.

Do you think that completing the fitness plan you read about would lead to better performance on this task?

- Yes
- No

If YES, how much improvement do you think would occur?

- 1 (a little)
- 2
- 3
- 4
- 5
- 6 (a lot)

This task measures how quickly and accurately someone can keep two different tasks in mind and switch back and forth between them. The primary measure of performance is the cost of having to switch between tasks. That is, how much slower are people when they have to switch tasks compared to when they keep doing the same task?

On each trial of this task, participants view a series of numbers and make one of two judgments about each one. The judgment they have to make depends on the color of the background behind the number. If the number appears against a blue background, participants judge whether it is greater than 5 or less than 5. If the background is pink, they judge whether the number is odd or even. They respond using the "z" key and the "/" key on their keyboard. The task compares their response speed when the have to make the same response two trials in a row to their response speed when they have to switch which response they are making.

Watch this video of a participant completing the task:

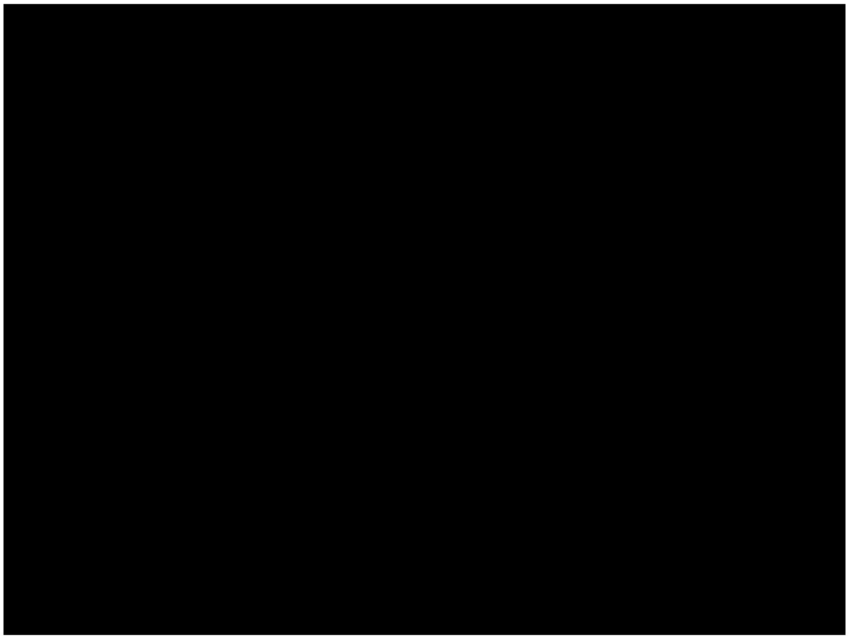

Briefly summarize the cognitive task you just learned about. Make sure to identify what a participant in that task must do and how their performance is measured. Please answer as best you can, even if you are not 100% certain.

Do you think that completing the fitness plan you read about would lead to better performance on this task?

- Yes
- No

If YES, how much improvement do you think would occur?

- 1 (a little)
- 2
- 3
- 4
- 5
- 6 (a lot)

Have you read or heard about research showing that exercise can improve cognition?

- ○ Yes
- ○ No

Please briefly describe what what you have read/heard about research showing that exercise can improve cognition:

Based on what you have heard or read, what types of exercise can improve cognition?

Do you exercise?

- ○ Yes
- ○ No

On average, how frequently do you exercise?

- Less than 1 time per week
- 1 time per week
- 2 times per week
- 3 times per week
- 4 times per week
- 5 times per week
- 6 times per week
- 7 times per week
- More than 7 times per week

What type or types of exercises do you do on a regular basis (at least once/week)

Are you male or female

- ◯ Male
- ◯ Female

What is your age in years?

[ ]

In what ZIP code do you live? (enter 5-digit ZIP code; for example, 00544 or 94305)

[ ]

Which option best describes your educational experience?

- ◯ Some high school
- ◯ High school graduate
- ◯ Some college (no college degree)
- ◯ Associate's degree (2-year college degree)
- ◯ Bachelor's degree (4-year college degree)
- ◯ Some graduate school
- ◯ Master's degree
- ◯ Professional degree
- ◯ Ph.D.

Thank you for completing this survey. You will have a chance to add comments when you return to Mechanical Turk. Before continuing, please enter a 5-digit number in the box below and copy it. To receive payment for the HIT, you will need to enter the same number when you return to the Mechanical Turk page. Please try to pick a number that will be unique to you.
